# Scraps

**Published:** 1899-06

**Authors:** 


### SCRAPS

PICKED UP BY THE ASSISTANT-EDITOR.

**Clinical Records** (27).—This excellent story has been recently revived :—"A well-known Archbishop of Dublin was, towards the end of his life, at a dinner given by the Lord-Lieutenant of Ireland. In the midst of the dinner the company was startled by seeing the Archbishop rise from his seat, looking pale and agitated, and crying, 'It has come—it has come!' 'What has come, your Grace?' eagerly cried half-a-dozen voices from different parts of the table. 'What I have been expecting for some years—a stroke of paralysis,' solemnly answered the Archbishop. 'I have been pinching myself for the last ten minutes, and find my leg entirely without sensation.' 'Pardon me my dear Archbishop,' said the hostess, looking up to him with a quizzical smile, 'pardon me for contradicting you, but it is my leg you have been pinching!'"

**Medical Satire.**—A friend has kindly supplied me with the following :—

Wisdom ceases not to be wisdom when it pleases her to assume the cap and bells of Folly; hence much profit can be derived from a perusal of the recently-issued "Comic Number" of the *Münchener medicinische Wochenschrift*, if readers, to borrow an expression from Rabelais, are wise enough "rompre l'os et sugcer la substantifique moelle."

Amongst the advertisements are to be seen: "Iodosanin," a new remedy for syphilis, wherein the iodine is so firmly secured no power on earth can release it; the organism is therefore protected against it; it is guaranteed to produce no iodism, *nor any other effect*.—Girls suffering from Dramatic Neurosis are thoroughly cured at the establishment of Schmierfink [Oily-tongue], Theatrical Manager, Vienna.—"Medical Assistant wanted for Hospital;" Salary £25 per annum, with board and lodging; a deduction of £22 15s. is made for attendance; liable to dismissal at a month's notice, but Assistant is otherwise bound for two years.—"Doctors required for a Club." Salary about 10d. a day; every other Good Friday a half-holiday; responsibility small, as every prescription is duly examined by the lay Committee, and if necessary corrected. Quacks and pot-house practitioners excluded, as the expense for soap and water would be too great.—"Exoteric Food for Invalids." This needs merely to be shown to the patients; strongly recommended, on the score of economy, to Hospitals and Infirmaries.—"Artificial Assistants." These are of glass and iron, with electrical and steam motive power; the surgical ones are transparent, sterilisable, call out "Lint," "Sponge," and then use them.

There is an original article by Dr. Michael Squirt on "The Artificial Production of Unsexual Generation in Man." Struck with Tolstoi's suggestion in the *Kreutzersonate*, that the actual method of generation, so crude, so coarse, is quite unworthy of our modern refinement and culture, the writer tries a lymph prepared by him from Protozoa that propagate by self-division. He experiments, by subcutaneous injection near the shoulder-blade, upon a wealthy banker, married, childless, eagerly desirous of an heir. Two heads soon form and complete themselves; but as each now claims to be the original banker and denounces the other for a scoundrel who is seeking to get possession of immense wealth and a handsome wife not his own, the dialogue grows animated. Finally two complete men are formed, to the utter bewilderment of the ever-watchful mother-in-law. Not to be outwitted, she too resolves on sub-division, and is injected accordingly. Division into four perfect individuals ensues. These now enter the bankers' mansion arm-in-arm. At the sight of the common enemy, the bankers blench with terror and collapse in a death-like swoon.—Dr. Pantschenberger's article, "A New Method of Feeding Infants," is admirable satire. The writer starts by condemning natural milk in every form, mother's milk included; maintains that modern Chemistry can in this matter excel Nature herself; and advances elaborate arguments in support of his thesis. He accordingly opens an experimental klinik with twenty-one infants, fourteen from three weeks to two months old, and seven from four to ten months These are fed with the doctor's artificial food exclusively. Results: Twelve out of fourteen of the former perish of inveterate stomachic affections; there are hopes three of the latter seven may survive the year. Now, the Professor's sunny optimism is by no means overclouded by these results, apparently so unfavourable; on the contrary, inasmuch as the preparation contains every element that is needful for the reparation and growth of the infantile organism, Nature is manifestly in error. "Let us but hold fast to Theory," says he, "Experience must and will give way!"—The article, "A Triumph of Modern Surgery," by Professor Swindel of Mississippi College, is aimed at the extravagant hardihood of Quixotic operators. The case is as follows: A young German, engaged for two and a half years in a paint-factory in America, falls ill. Symptoms: Several times a day the stomach, after premonitory noises—rolling, gurgling, gulping,—discharges its fluid contents. Great thirst supervenes, but the drinking of fluids only aggravated the evil. As no treatment relieves, Dr. Swindel boldly determines to excise the stomach and substitute a swine's. The operation is performed. Then in the stomach is discovered the cover of a beer jug, swallowed in student days and long forgotten. Complete success. Healing *per primam.* In the exuberance of restored health, the young man shortly afterwards climbs a lamp-post, which snaps off and flings him to the ground, the lamp descending with terrific force upon his occiput and causing compound fracture of the skull. Brought almost *in articulo mortis* into Dr. Swindel's klinik, a hasty examination reveals the skull-cavity all but empty, the brain-matter having been lost through the wound. As a desperate resort, a calf's brain is planted therein, with the happiest results. The patient recovers: every mental operation is soon normal. He quits the paint-business and turns art-critic with marked distinction. "This brilliant success," says the writer, "secures for brain-transplantation an enduring place in surgery."

New Books received:—

Alois Strampfer. *The Most Common Forms of Injury arising from Cycling—to Foot-passengers.* Leipzig. 1898.

G. Sorcier. *Acceleration of the Mental Development of New-born Infants by Inoculation with Extract from the Pituitary Gland.* Paris. 1898.

John Grudger. *The Morbid Sensibility of the English—particularly in Reference to the Naval Advancement of Other Nations.* London. 1898.

Paolo Firlefanzi. *The Voluntary Procreation of Twins.* Firenze. 1898.

Contributions in verse occupy about half the number. Of these, the best perhaps is the short epigram, "The Mistaken Examination," concerning a physician whose custom is to look for fever *per anum.*

At the sitting of the Medical Association in München, November 31, 1898, Dr. Masculine proposed certain addenda to the usual code of professional etiquette, which, legislating for men only, is now obsolete. Among them are these: The male consultant is on no account to assume airs of superiority over the Doctoress, nor presume to criticise the treatment followed. Children of a Doctor and Doctoress may choose by a bare majority which parent is to take medical charge of them. In case of illness occurring in the medical husband or wife, either may give medical aid to the other gratis. This rule extends to childbirth. But if the latter event is too often repeated, the husband's fees must be settled by mutual arrangement.

**Medical Philology** (XXX.).—The *Promptorium* has "DYDERYN' for colde. *Frigucio, rigeo,*" and "DYDERYNGE, *Frigitus.*" Mr. Way's note quotes "dadir" from the *Catholicon Anglicum,* and from Cotgrave "*Barboter de froid,* to chatter or didder for cold, to say an ape's Paternoster;" and adds, "Skinner gives this word as commonly used in Lincolnshire, '*a Belg.* sitteren, *præ frigore tremere.*' The Medulla renders '*frigucio,* romb for cold.' In the Avowynge of King Arther, edited by Mr. Robson, to 'dedur' has the sense of shaking, as one who is soundly beaten; and in the Towneley Mysteries, Noah's wife, hearing his relation of the approaching deluge, says,

'I dase and I dedir

For ferd of that taylle.' p. 28.

Didder, to have a quivering of the chin through cold' FORBY."

Mr. Herrtage, in his edition of the *Catholicon*, has a note on "to dadir" (the Latin equivalent of which is *Frigucio*), saying: "*Dither* is still in use in the Northern Counties with the meaning of 'to shake with cold, to tremble': see Peacock's Gloss. of Manley & Corringham, Nodal's Glossary of Lancashire, &c. *Dithers* is the Linc. name for the shaking palsy, *paralysis agitans*. The Manip. Vocab. gives 'to dadder, *trepidare*.' Cotgrave has '*Claquer les dents*. To gnash the teeth, or to chatter, or didder, like an Ape, that 's afraid of blowes. *Frisson*. A shivering, quaking, diddering, through cold or feare; a trembling or horror.' See also *Friller*, *Frissoner*, and *Grelotter*.

> Boyes, gyrles, and luskyth strong knaves,

> *Dyddderyng* and *daddcryng* leaning on ten staves.'

> *The Hye way to the Spyttel Hous*, ed. Hazlitt, p. 28."

Didder and dither are fairly common in colloquial speech even now. The *New English Dictionary* quotes "ditthering, rippling hysteria," from Rudyard Kipling's *Soldiers Three*.

Mr. Herrtage, calling attention to its connection with "Dayse" (which in the *Catholicon* has the meaning "to be callde" with *frigere* as one of its Latin renderings), in his note has: "Icel. *dasdr*, faint, tired; *das*, a faint, exhaustion."

The *Catholicon* has "Calde of þe axes; *frigor*." This is interestingly illustrated by Mr. Herrtage, who says "Palsgrave gives 'Chyueryng as one dothe for colde. In an axes or otherwise, *frilleux*. Ague, axes, *fyeure*.' *Axis* or *Axes* is from Lat. *accessum*, through Fr. *accez*, and is in no way connected with A.S. *æce*. Originally meaning an approach or coming on of anything, it at an early period came to be specially applied to an approach or sudden fit of illness: thus Chaucer has, 'upon him he had an hote *accesse*.' *Black Knight*, l. 136, and Caxton, 'fyl into a sekenes of feures or *accesse*.' *Paris & Vienne*, p. 25." *The Complaint of the Black Knight* is not now considered to be Chaucer's work, but the word in this sense occurs in his undoubted *Troilus and Criseyde*:

> "A charme that was right now sent to thee,
> The whiche can thee hele of thyn accesse." II. 1314–15.

> "What nedeth you to tellen al the chere
> That Deiphebus unto his brother made,
> Or his accesse, or his sikly manere." II. 1541–43.

Under "Axes," which in the *Catholicon* has a reference to fevers, Mr. Herrtage has this note: "In the Paston Letters, iii. 426, we read—'I was falle seek with an *axez*.' It also occurs in *The King's Quhair*, ed. Chalmers, p. 54:

> 'But tho begun mine *axis* and torment,'

with the note—'*Axis* is still used by the country people, in Scotland, for the ague.' Skelton, Works, i. 25, speaks of

> 'Allectuary arrectyd to redres These feverous *axys*.'

'Axis, Acksys, aches, pains.' Jamieson. 'I shake of the axes. *Je tremble des fieures*.' Palsgrave. 'The dwellers of hit [Ireland] be not vexede with the *axes* excepte the scharpe axes [incolæ nulla febris specie vexantur, excepta acuta, et hoc perraro]. Trevisa, i. 333. See *Allit. Poems*, C. 325, '*þacces* of anguych,' curiously explained in the glossary as blows, from A.S. *þaccian*." The *New English Dictionary* quotes from Andrew, who, in his 1527 translation of *Brunswyke's Distyll. Waters*, recommends a certain treatment "for the dayly axces or febres." Jamieson, in the passage here quoted from him, seems to have fallen into the error of interpretation against which Mr. Herrtage warns us in the note on "Calde of þe axes" given above. Concerning the different pronunciations of "ache" as a noun and a verb, I had a long note in the *Journal* for June, 1892. This is a word which we get from the Anglo-Saxon, *æce* or *ece*, a pain or unpleasant feeling.

"To say an ape's Paternoster," which occurs in Mr. Way's note above, was a proverbial expression for chattering with cold, and probably had some reference to the noise of the beads in the Rosary at every tenth of which a Paternoster was said. In addition to the instances in Cotgrave to which Mr. Way and Mr. Herrtage refer, Halliwell, in his *Dictionary of Archaic and Provincial Words*, gives Batre, Cressiner, and Dent. Under the first of these Cotgrave has "Batre le tambour avec les dents. *To chatter, didder, say an apes Paternoster*."

